# Achieving Successful Pregnancy in a Patient With Ovarian Endometriosis Through Assisted Reproductive Technology (ART) Intervention: A Case Report

**DOI:** 10.7759/cureus.53014

**Published:** 2024-01-26

**Authors:** Prerana Dagwar, Akash More, Namrata Choudhary, Jarul Shrivastava, Princee Tyagi

**Affiliations:** 1 Clinical Embryology, Datta Meghe Institute of Higher Education and Research, Wardha, IND

**Keywords:** estradiol, art, fertility treatment, infertility, ovarian endometrioma

## Abstract

Endometriosis stands out as the predominant issue among gynecological disorders. Approximately 50% of women experiencing infertility have been observed to be affected by endometriosis. Growing evidence indicates that endometriomas, in isolation, might adversely affect ovarian physiology. Medications such as ethinyl estradiol and levonorgestrel have been found to contribute to the amelioration of ovarian cysts in women of reproductive age. Presently, there are limited alternatives available for addressing the infertility associated with endometriosis. The primary effective approaches for treatment still revolve around surgery and assisted reproductive technology for endometriosis-related infertility. This case study centers on the utilization of ethinyl estradiol and levonorgestrel in patients with ovarian endometrioma, leading to an enhancement in the likelihood of achieving a successful clinical pregnancy.

## Introduction

Endometriosis is the presence of endometrial tissue outside of the uterus. It can affect many reproductive organs, including the pelvic area, fallopian tubes, and ovaries, frequently resulting in symptoms such as infertility, dysmenorrhea, dyspareunia, and chronic discomfort [1]. Infertility is a condition in which the couple is unable to conceive even after a period of continuous, unprotected sexual mating of 12 months or more [2]. The most prevalent issue among gynecological illnesses is endometriosis. The most common locations where endometriotic lesions form are the pelvic peritoneum and ovaries [3]. Endometriotic lesions can also arise in the intestines, cervix, fallopian tubes, bladder, abdominal wall, and vagina. Many gynecologists posit that inflammation is crucial in causing discomfort in endometriosis, even though the exact pathogenesis of endometriosis-related pain is not understood [3]. Ovarian endometriomas are differentiated by the appearance of endometrial tissue within the ovary [4]. Around 15-44% of women with endometriosis are not aware of the presence of ovarian endometriomas, and in 19-28% of cases, both ovaries are affected [1]. There are currently few alternatives for treating endometriosis-related infertility. Effective therapy revolves around surgery and assisted reproductive technology (ART) [5].

Certain medicines, like ethinyl estradiol and levonorgestrel, help in the improvement of ovarian cysts in reproductive-age women. This case study revolves around the use of ethinyl estradiol and levonorgestrel in patients with ovarian endometrioma, thus improving the chances of a successful clinical pregnancy.

## Case presentation

A 29-year-old female and her 30-year-old spouse were recommended to a fertility center and visited a fertility clinic to address infertility concerns after two years of marriage. The patient reported that she used to have lower back pain and cramps a few days before her menstrual period, which extended a few days even after her menstrual period. The patient used to take painkillers before, during, and after her menstrual cycle whenever she felt uneasy. After two years of marriage and regular intercourse, the couple was unable to conceive. The female patient was diagnosed with ovarian endometrioma in both ovaries, but no past treatment was taken. The couple sought infertility treatment for the first time. The woman had a body mass index (BMI) of 22 Kg/m^2^, and her husband's BMI was 23 Kg/m^2^, falling within the normal range as the recommended BMI range for most adults is between 18.5 Kg/m^2^ and 24.9 Kg/m^2^.

**Figure 1:**
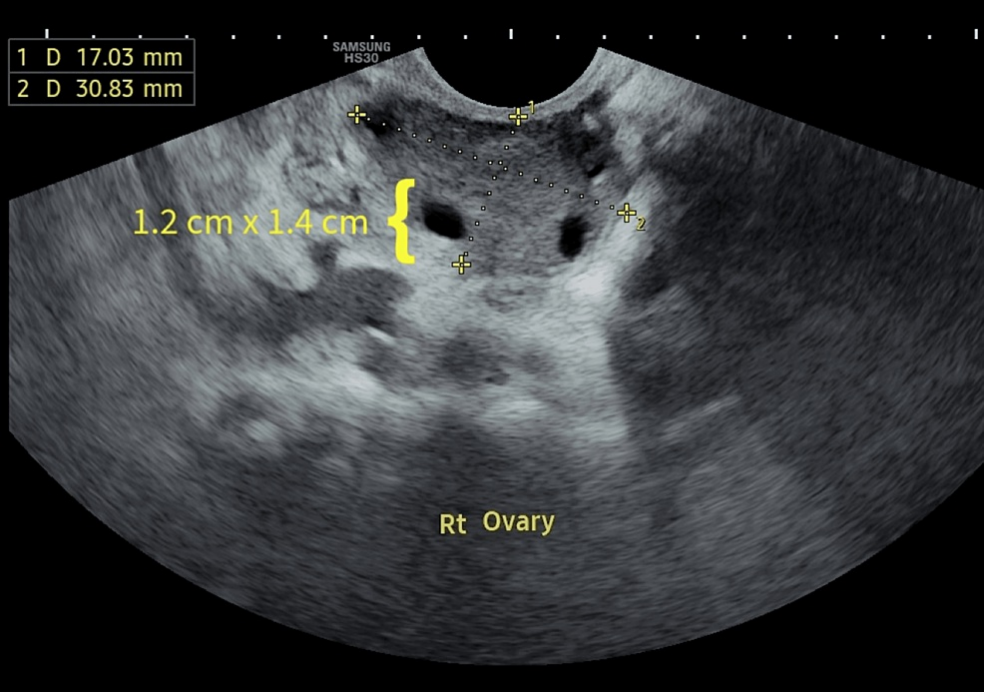
Day 2 transvaginal sonography of the patient's right ovary

**Figure 2:**
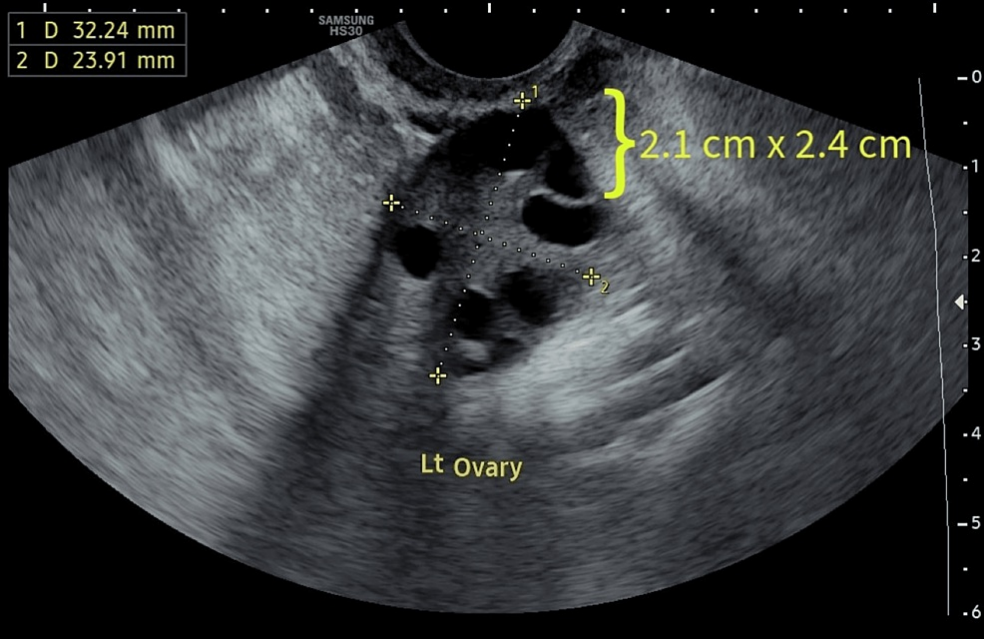
Day 2 transvaginal sonography of the patient's left ovary

The woman and her husband underwent an infertility assessment to identify the underlying cause of their fertility challenges. The husband’s semen analysis revealed asthenozoospermia, progressive motility of less than 30% as per the lower reference limit mentioned in the World Health Organization (WHO) manual's sixth edition [6]. The male patient's hormone evaluation revealed normal levels of prolactin, luteinizing hormone (LH), testosterone, and follicle-stimulating hormone (FSH). The female patient underwent a transvaginal ultrasound (TVS). Figure 1 indicates day 2 transvaginal sonography of the patient's right ovary that shows an intraovarian cystic lesion with isoechoic texture and peripheral echogenic foci. It measures 1.2 x 1.4 cm and Figure 2 indicates day 2 transvaginal sonography of the patient's left ovary. It revealed that the left ovary showed one large cystic lesion measuring 2.1 cm x 2.4 cm with homogeneous diffuse hypoechoic fluid content and fine low-level echoes; the cysts from the right ovary seen in Figure 1 increased to 2.9 cm x 2.6 cm and the cyst in the left ovary increased to 4.1 x 3.9 cm on day 8 of menstruation. It also shows a dependent echogenic focus. The female partner underwent a transvaginal ultrasound to evaluate her ovarian reserve and examine for any potential structural abnormalities. On the right ovary, two follicles were observed, while the left ovary displayed one follicle during the ultrasound examination.

In this case, infertility is the primary type. The probable cause of infertility was a large ovarian cyst affecting the menstrual cycle as well as the quality of oocytes and asthenozoospermia in her husband.

Treatment

Originally, the patient underwent controlled ovarian stimulation to increase the number of viable eggs available for fertilization. We employed the GnRH (gonadotropin-releasing hormone) antagonist protocol to induce ovarian stimulation for the oocyte retrieval procedure in the female. She commenced a daily routine of taking 2 mg of estradiol (E2) tablets twice a day, along with vitamin supplements. Additionally, as part of pre-treatment medication, she was prescribed a once-daily thyroxine supplement tablet of 150 mcg. Following that, ovarian stimulation was initiated using gonadotropins. The ovarian response was assessed through a combination of ultrasound scans and serum estradiol measurements. The trigger was administered once the follicles reached the desired size of 18 mm and above. Following this, we administered a subcutaneous injection of 10,000 IU of human chorionic gonadotropin (hCG) to the patient, responsible for inducing oocyte maturation. We attempted transvaginal ovum pick-up 36 hours after administering the injection, leading to the retrieval of three oocytes. Nevertheless, two of them were germinal vesicle (GV) oocytes, and the third was a metaphase I (MI) oocyte. After seven hours, the MI oocyte progressed to maturity, becoming a metaphase II (MII) oocyte. Intracytoplasmic sperm injection (ICSI) was conducted on the MII oocyte, but it degenerated on the second day.

**Figure 3:**
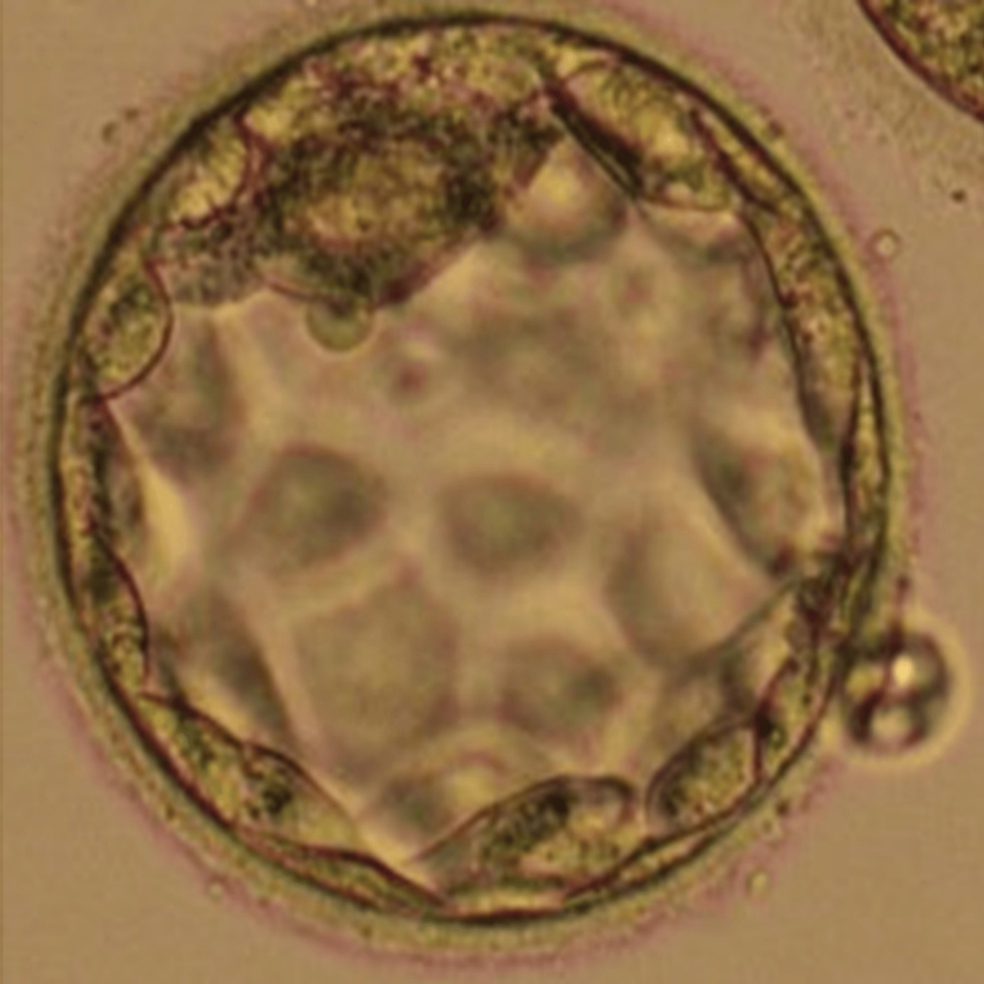
Day 5 blastocyst stage embryo transfer

After that, levonorgestrel 0.1mg, ethinyl estradiol 0.02mg, and ethinyl estradiol 0.01mg were prescribed on the first day after the onset of menstruation. As menstruation began, one ethinyl estradiol 0.02 mg/levonorgestrel 0.1 mg tablet was taken that day. For each 91-day course, medication was taken in the following order: one ethinyl estradiol 0.02 mg/levonorgestrel 0.1 mg tablet was taken daily for 84 consecutive days. Then one ethinyl estradiol 0.01 mg tablet was taken for seven consecutive days. A scheduled period had occurred during the seven days, so the ethinyl estradiol 0.01 mg tablets were taken. Three months later, we attempted another transvaginal ovum pick-up 36 hours after administering the injection, leading to the retrieval of five oocytes. Nevertheless, three of them were MI oocytes, and the remaining two were in MII. Two hours later, we conducted ICSI on the MII oocyte, leading to the development of a high-quality blastocyst by day 5. On the 18th day, we conducted a single day 5 blastocyst stage fresh embryo transfer as shown in Figure 3, and the procedure was well-tolerated by the patient.

Follow up

After the embryo transfer went well, the patient was released from the hospital with instructions for continued care. The patient was prescribed a course of treatment that included two tablets per day of ofloxacin and ornidazole, 40 mg of omeprazole on an empty stomach, a daily vitamin E supplement, one tablet of arginine per day, general vitamin supplements, two tablets of estradiol (2 mg) per day, and one 400 mg dose of progesterone per day. She was also advised to take 500 mg of hydroxyprogesterone, injectable human chorionic gonadotropin (hCG), and intrepid injections. Following that, both the report and the β-hCG test came back positive. The recorded concentration of β-HCG was 1050 mIU/ml.

## Discussion

One typical and benign sign of endometriosis is the histological development of functional endometrial glands or stromal endometriosis outside the uterine cavity. It has been shown to affect about 50% of infertile women [7]. Ovarian endometriomas, also called chocolate cysts, were found to be most prevalent in reproductive-aged women [4]. Ovarian endometriosis is found to be present in up to 30%-40% of women with endometriosis [7]. Research has shown that fibrosis in the ovarian cortex reduces ovarian function and follicles in 55% of women with ovarian endometrioma [8]. Ovarian endometrioma rarely exceeds 6 cm in diameter. Endometrioma of the ovaries larger than 10 cm in dimension is known as giant endometrioma [4]. The size of the endometrioma is the main factor affecting the overall number of follicles and oocyte recovery. The expected number of oocytes recovered falls by 0.667 for each milli-meter of increased endometrioma size, all equal [1].

Our results are in line with the research by Conti et al., which showed a higher risk of preterm birth (PTB) and preterm pre-labor rupture of membranes. Their research also revealed increased risks of being admitted to the Neonatal Intensive Care Unit (NICU) and having babies born short for gestational age [9]. One hallmark of endometriosis is persistent intraperitoneal inflammation [2]. Infertility in endometriosis may be impacted by persistent inflammation in several ways. Increased levels of interleukin-1b (IL-1b), IL-8, IL-10, and tumor necrosis factor-a in follicles next to endometriomas are associated with reduced ovarian response [10]. It is believed that progesterone resistance in endometriosis-affected women causes deregulation of genes crucial for embryo implantation, which may ultimately result in miscarriage [4]. In two further recent meta-analyses of the process' results, Patients with alternative In vitro fertilization (IVF) indications and minimal/mild endometriosis had comparable rates of live births. In contrast, patients with moderate or severe endometriosis showed worse outcomes, such as fewer oocytes retrieved, a lower implantation rate, and a lower birth rate [10].

To date, TVS has a high sensitivity (93%) and specificity (97%) for detecting ovarian endometriomas when used by a skilled operator. Several studies have examined the reproductive outcomes following surgery for ovarian endometriomas [1]. There is a chance of ovarian damage when endometriosis is surgically treated. Surgery as a therapy option has raised significant concerns about this, and numerous attempts have been made to reduce the ovarian tissue damage that is linked with it [11]. A worse reproductive prognosis with a higher risk of premature ovarian failure could result from this injury, even if the skilled surgeons provided consistent and cautious therapies. A single study specifically assessed the likelihood of spontaneous pregnancy in individuals with endometriomas who did not have a prior history of infertility. Increasing evidence suggests that endometriomas, in and of themselves, may harm ovarian physiology. According to a systematic review by Sanchez and colleagues, the existence of an endometrioma is implicated in ovarian damage, irrespective of its size [12].

Mechanical elements, including stretching, compressing healthy tissue, and obstructing normal blood flow, are blamed for this damage. Proteolytic enzymes, inflammatory chemicals, reactive oxygen species, iron, and other components that lead to cellular damage are found in higher concentrations in endometriomas. The unequal distribution of antioxidant and oxidant compounds in FF and serum has been suggested as a possible reason for abnormal oocyte development. This imbalance may lead to DNA and cell membrane damage, impacting fertilization, implantation, and embryonic development, ultimately resulting in diminished egg and embryo quality [1]. Amenorrhea, irregular menstrual cycles, and dysmenorrhea can all be effectively treated with ethinyl estradiol and levonorgestrel tablets. Additionally, they speed up the elimination of functional ovarian cysts and are linked to excellent success rates for those who have them [13]. When opting for conservative management for patients with endometriomas greater than 3 cm and moving forward with IVF, it is crucial to consider two factors: first, the number of women with large endometriomas undergoing IVF-ICSI is not high; and second, endometriomas larger than 7-8 cm carry an increased risk of infection and cancer from pick-up [1]. Patients with endometriosis who are infertile can successfully increase their pregnancy rate through in vitro fertilization and embryo transfer (IVF-ET). However, compared to other patients, patients with endometriosis had much poorer pregnancy results following IVF-ET [8].

## Conclusions

In summary, ovarian endometrioma has an impact on ovarian functions. This leads to infertility in patients with ovarian endometrioma and reduces the chances of a successful pregnancy. Even with ovarian endometrioma, a successful pregnancy can be achieved through treatment with ART.
